# Consumer-Grade Neurofeedback With Mindfulness Meditation: Meta-Analysis

**DOI:** 10.2196/68204

**Published:** 2025-04-17

**Authors:** Isaac Treves, Zia Bajwa, Keara D Greene, Paul A Bloom, Nayoung Kim, Emma Wool, Simon B Goldberg, Susan Whitfield-Gabrieli, Randy P Auerbach

**Affiliations:** 1 McGovern Institute for Brain Research Massachusetts Institute of Technology Cambridge, MA United States; 2 Department of Brain and Cognitive Sciences Massachusetts Institute of Technology Cambridge, MA United States; 3 Department of Psychology University of California Berkeley, CA United States; 4 Center for Precision Psychiatry Massachusetts General Hospital Boston, MA United States; 5 Department of Psychiatry Columbia University New York City, NY United States; 6 Department of Counseling Psychology University of Wisconsin–Madison Madison, WI United States; 7 Department of Psychology Northeastern University Boston, MA United States; 8 Division of Child and Adolescent Psychiatry New York State Psychiatric Institute New York, NY United States

**Keywords:** neurofeedback, mindfulness, device, neurotechnology, meditation, stress, cognition, Muse, consumer grade, meta-analyses, neuroscientific technology, neurotechnologies, randomized

## Abstract

**Background:**

There is burgeoning interest in the application of neuroscientific technology to facilitate meditation and lead to beneficial psychological outcomes. One popular approach is using consumer-grade neurofeedback devices to deliver feedback on brain targets during meditation (mindfulness-based neurofeedback). It is hypothesized that optimizing brain targets like alpha and theta band activity may allow meditators to experience deeper mindfulness and thus beneficial outcomes.

**Objective:**

This study aimed to systematically review and meta-analyze the impacts of consumer-grade mindfulness-based neurofeedback compared with control conditions. Included studies involved mindfulness practice operationalized as open monitoring or focused attention meditation. This study was preregistered.

**Methods:**

A total of 16 randomized controlled training trials, as well as 5 randomized within-participant designs were included, encompassing 763 and 167 unique participants, respectively. Effects were categorized outcomes (ie, psychological distress, cognitive function, and physiological health) and process variables (ie, state mindfulness and brain measures). Study risk of bias, reporting bias, and publication bias were assessed.

**Results:**

Samples were typically small (n=30-50), and the majority of studies used mindfulness apps as controls. To deliver neurofeedback, most studies used the Muse device (11/16 randomized controlled trials [RCTs]). There was a modest effect for decreases in psychological distress compared with controls (*k*=11, *g*=–0.16, *P*=.03), and heterogeneity was low (*I^2^*< 0.25). However, there was no evidence for improvements in cognition (*k*=7, *g*=0.07, *P*=.48), mindfulness (*k*=9, *g*=0.02, *P*=.83), and physiological health (*k*=7, *g*=0.11, *P*=.57) compared to controls. Mechanistic modulation of brain targets was not found in RCTs or within-participant designs. Sex (male or female), age, clinical status, study quality, active or passive controls, sample size, and neurofeedback duration did not moderate effects. There was some evidence for reporting bias, but no evidence of publication bias. Adverse effects were not assessed in 19 out of 21 studies and not found in the 2 studies that assessed them.

**Conclusions:**

Assertions that consumer-grade devices can allow participants to modulate their brains and deepen their meditations are not currently supported. It is possible that neurofeedback effects may rely on “neurosuggestion” (placebo effects of neurotechnology). Future research should examine more extensive calibration and individualization of devices, larger sample sizes, and gold-standard sham-controlled RCTs.

## Introduction

Mindfulness meditation involves cultivating nonjudgmental attention to experiences in the present moment [1]. Mindfulness-based interventions (MBI) are an increasingly popular means of promoting well-being, and there is systematic evidence of benefits for children, adolescents, adults, and older adults [2-4]. They are used in nonclinical and clinical populations [5]. For mental health disorders like anxiety, MBIs have been documented to be equally effective as pharmacological treatment [6]. Traditional MBIs involve in-person group training led by experienced teachers. Such MBIs are not easily scaled, teacher training is largely nonstandardized, and some participants may be resistant to group settings [7]. An alternative is technology-supported mindfulness, an umbrella term encompassing mobile apps [8], virtual reality and augmented reality [9], video games [10], biofeedback [11], and neurofeedback [12,13]. Here we systematically review and meta-analyze mindfulness-based neurofeedback (mbNF) of consumer-grade neurofeedback devices to understand its effectiveness.

One of the most commonly used MBIs is mindfulness-based stress reduction (MBSR) [14]. MBSR consists of 8 weeks of group classes and meditation training, with a full-day retreat in the sixth week. Meditations include practices like breath awareness, which involve orienting attention to one’s breath and practicing returning to the breath every time one’s attention wanders away, and body scans, which involve moving the spotlight of attention from body part to body part with a curious and nonjudgmental attitude toward the sensations that one encounters. The MBSR program recommends 45 minutes of practice per day, although true adherence is often substantially less [15,16]. Overall, the goal of MBSR is to teach participants to become more aware of their experiences in the present moment, and to cultivate a nonjudgmental, accepting attitude toward those experiences.

Mindfulness interventions like MBSR have been shown to decrease anxiety, stress, and negative affect [17-22]. Interventions may also lead to increased sense of life’s meaning and purpose [23]. Positive cognitive outcomes have also been observed. Along with sustained attention [24,25], MBIs may benefit working memory and other executive functions [26-30]. Thus, MBIs have been associated with both positive cognitive outcomes and decrease in psychological distress. One reason is that repeated meditation practice may facilitate mindful dispositions outside of practice; indeed, this “trait” mindfulness often increases in MBIs [31].

MBSR and other teacher-led, in-person mindfulness interventions are beneficial but relatively hard to scale. This has led to a proliferation of technology supported mindfulness [8-13]. Neurofeedback may be one method of supporting or enhancing mindfulness learning [12,32,33]. Neurofeedback consists of measuring brain signals during a task and relaying those signals (targets) to the participant. The participant may learn with repeated practice to modulate the target, with beneficial outcomes. The primary proposed mechanism of neurofeedback is that it involves facilitating the learning of strategies or skills [34,35]. In the context of meditation, a practitioner may want to learn how to attend mindfully to the breath and regulate mind-wandering or distractions. Neurofeedback may be relayed through auditory or visual stimuli. As the participant becomes calmer and more focused (assuming this may be detected by brain signals), the stimuli relayed to them are systematically altered. With practice the participants can learn to alter the stimuli. In summary, neurofeedback has been proposed to act as a “technological mirror” reflecting the intricacies of the mind back to the practitioner [36]. We call this application of mbNF.

Neurofeedback may be conducted in the laboratory, with techniques like electroencephalography (EEG) and functional magnetic resonance imaging (fMRI), or in the real world with consumer-grade devices. Laboratory-based neurofeedback generally has been found to aid in learning attentional skills, emotion regulation skills, and pain management skills [37-43]. Yet, neurofeedback research has been criticized for inadequate control conditions and other methodological shortcomings [44,45]. In a recent systematic review, we examined the state of the evidence in laboratory mbNF [13]. While the studies were too heterogeneous to conduct a formal meta-analysis, we identified that mbNF shows promise for improving hard-to-change clinical symptoms as well as increasing state mindfulness. It is possible that these effects are driven by participants learning to regulate brain targets. Becoming aware of experiences in the present moment may lead to decreases in activation in the default-mode network (fMRI), involved in self-referential thinking and rumination [13]. Likewise, mbNF may allow participants to upregulate frontal midline theta oscillations (EEG), which are related to states of inward attention [46]. However, we also identified that many studies lacked gold-standard control conditions, and reporting standards were not met.

**Figure 1:**
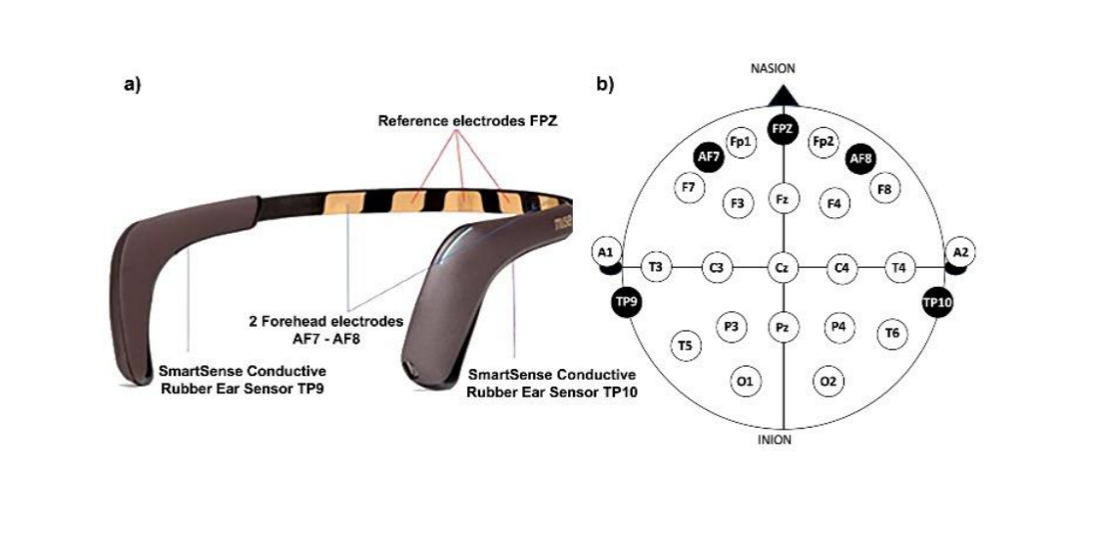
Visual representation of consumer-grade device Muse. (A) Muse 2 headband sensors overview. (B) Top-down view of the electrography electrode positions on the subject’s head according to the 10-20 system, standard for electrography recordings. The device includes two forehead electrodes (AF7 and AF8), reference electrodes at FPz, and conductive rubber ear sensors at TP9 and TP10. The FPz electrode serves as a reference point and is positioned at the midline of the forehead. The nasion is the anatomical landmark at the bridge of the nose, used for consistent electrode positioning, while the inion is the bony prominence at the back of the skull. Obtained from Mansi et al [47], published under Creative Commons Attribution 4.0 International License [48].

Consumer-grade neurofeedback (cgNF; which relies on small sets of dry electrodes, Figure 1 [47,48] and Textbox 1) is much cheaper and easier to implement at scale than laboratory mbNF [49]. There are some promising initial indications for the effectiveness of cgNF for executive functioning [50,51]. However, the evidence base for cgNF is scarce [52], especially in the realm of meditation, where the enthusiasm for these devices may outpace the evidence [53]. For example, the Muse headband, which markets itself as “your personal meditation coach,” offers to elevate mental performance, improve sleep, improve focus, and more (InteraXon). As yet, there is no systematic evidence that devices like Muse can effectively promote these psychological and physiological benefits. Despite this, Muse reports over 500,000 users. Motivated by the enthusiasm-evidence gap and by the potential of new frontiers in technology-supported mindfulness, the aim was to systematically review and meta-analyze cgNF applications for mindfulness.

**Brief description of consumer-grade neurofeedback device protocols.**

Among the consumer-grade devices commonly used for mindfulness-based neurofeedback is Muse, a portable dry electrocardiography (EEG) system. Dry EEG systems like Muse detect brain activity through sensors that do not require conductive gels, making them convenient for real-world use. Muse features sensors on the forehead (AF7 and AF8) and behind the ears (TP9 and TP10) to detect electrical signals generated by brain activity. These signals are amplified, filtered, and transmitted through Bluetooth to a connected device, where they are analyzed in real time or stored for later use. Participants engage in a calibration before mindfulness-based neurofeedback, which serves as a baseline for algorithms that compute brain states. Calibration is a common part of neurofeedback protocols as it allows the algorithms to detect personalized differences between targeted and alternative brain states like rest [13]. The implementation varies widely across consumer-grade devices. For example, in early versions of Muse a word-association task was used, but in Muse 2.0 a brief body scan meditation is used. Unfortunately, calibration procedures were not systematically reported in the studies analyzed here. Although algorithms are private due to proprietary concerns, it is likely that Muse uses an alpha and theta training model, rewarding alpha waves (related to attention), and theta waves (related to relaxation). The model may involve additional EEG frequencies like delta and beta, while an artifact correction algorithm is used to remove muscle and ocular activity [56-59]. Signals are classified into “Active,” “Neutral,” and “Calm” states by the Muse app and relayed to the participant using auditory feedback (eg, ocean sounds) during the meditation session. Other systems include Emotiv, with 14 electrodes, Lowdown Focus [smith optics], which has 5 electrodes and resembles eyewear, and Omni [omni neurofeedback], with 2 electrodes.

It should be noted that typical cgNF studies compare neurofeedback training with the devices with training with a comparison condition, often mobile apps. Briefly, app-based mindfulness programs, which involve progressing through a series of recorded meditations, can show many of the same benefits as traditional mindfulness [60,61], but with smaller effect sizes [62,63]. One reason may be lack of adherence [64,65]. Another reason may be the lack of teachers, who provide social support and help participants learn practices through expert feedback [66]. In the current context, cgNF may offer the additional support and incentive necessary for more effective mindfulness learning.

For the present study, 2 types of randomized studies were included. We leveraged randomized controlled trials (RCTs) with between-participant controls as well as within-participant controls (ie, participants perform mbNF and meditation only, with the order randomized). The following open questions were addressed using quantitative synthesis: (1) Is there evidence that participants are learning to regulate brain signals using neurofeedback, and reporting higher mindfulness? (2) Are there benefits of neurofeedback compared with control conditions? In terms of beneficial outcomes, we collected evidence in the domains of psychological distress, cognition, and physiological health. In addition, we examined methodological limitations (eg, the possibility of placebo effects) and possible moderators like clinical conditions. Our meta-analysis is the first systematic evaluation of whether cgNF with mindfulness meditation enhances outcomes and processes related to mindfulness.

## Methods

### Objectives

The objective of this meta-analysis was to assess whether to mindfulness meditation concurrent with consumer-grade neurofeedback had beneficial effects compared with control conditions. Mindfulness meditation is known to have benefits for psychological distress, cognitive functioning, and physiological health [3,67-69], and we assessed whether mbNF had significantly more positive outcomes in these domains. Separately from outcomes, we also assessed effects of mindfulness on process variables like state and trait mindfulness, as well as whether participants can modulate brain signals through mbNF. We preregistered this meta-analysis before examination of the data, on OSF Registries [70]. All deviations can be found in Multimedia Appendix 1.

### Eligibility Criteria

Randomized studies that used cgNF (Figure 1 [47]) concurrently with mindfulness meditation and had a control condition were included (Textbox 2 and Table 1).

**Inclusion and exclusion criteria for mindfulness-based neurofeedback studies.**

**Inclusion criteria**

- Article type: papers, dissertations, chapters, and preprints.
- Language: English.
- Consumer-grade neurofeedback: a device that recorded scalp electroencephalograph (EEG) in real time, gave the user feedback in real time based on the recorded EEG signal, and was commercially available to the public (Figure 1 [47]).
- Meditation: mindfulness practices such as focused attention or open monitoring [54,55,71].
- Concurrent neurofeedback and mindfulness meditation: neurofeedback and mindfulness meditation occurred at the same time.
- Control condition: included control condition (between-person or within-person).
- Randomization: included randomization of groups or condition.
- Participant demographics: all ages and populations.

**Exclusion criteria**

- Article type: nonempirical status (eg, reviews and meta-analysis).
- Consumer-grade neurofeedback: non-commercially available to public (eg, laboratory-based EEG).
- Meditation: other meditations (eg, transcendental and loving kindness and compassion) or no meditation.
- Randomization: no randomization.

**Table 1:** Table1. Overview of all randomized controlled trails included in the meta-analysis.

Study identifier	Participant information	Participant number	Condition	Meditation	Outcome types
Acabchuk et al [53]	20.52 years, 73.1% F^a^, NT^b^	25 27	Muse Active (MAU appb)	FA^c^, 4 weeks	Brain target, mindfulness, psychological distress
Balconi et al [72]	NR^d^, NR, NT	20 20	LFe Active (MAU app)	FA, 4 weeks	Cognitive, mindfulness, psychological distress
Balconi et al [73]	24.2 years, 76 % F, NT	25 25	LF Active (MAU app)	FA, 3 weeks	Cognitive, mindfulness, physiology
Balconi et al [74]	NR, NR, NT	18 17	LF Active (MAU app)	FA, 4 weeks	Physiology, psychological distress
Balconi et al [75]	23.12 years, 69.09% F, NT	28 27	Muse or LF Active (MAU app)	FA, 4 weeks	Physiology, psychological distress
Balconi and Crivelli [76]	23.58 years, NR, NT	19 19	LF Active (MAU app)	FA, 4 weeks	Cognitive, mindfulness, physiology, psychological distress
Bhayee et al [77]	32.65 years, 46.16 % F, NT	13 13	Muse Active (math education)	FA, 6 weeks	Cognitive, mindfulness, psychological distress
Crivelli et al [78]	22.94 years, NR, NT	17 18	Muse Active (MAU app)	FA, 2 weeks	Cognitive, mindfulness, psychological distress
Crivelli et al [79]	23.47 years, NR, NT	18 18	Muse Active (MAU app)	FA, 4 weeks	Cognitive
Hawley et al [80]	26 years, NR, OCD^f^	25 24	Muse Passive	unclear, 8 weeks	Mindfulness, psychological distress
Min et al [81]	38.64 years, 90.22% F, NT^g^	30 63	Omni Active (MAU app/ self-care)	FA/body scans, 4 weeks	Brain target, mindfulness, physiology, psychological distress
Polich et al [58]	45.4 years, 85% F, TBI^h^	10 10	Muse Active (MAU app)	FA, 6 weeks	Brain target, cognitive, mindfulness, psychological distress
Schuurmans et al [82]	14.46 years, 40% F, PTSD^i^	8 3	Muse/MindWave Active (breathing game)	OM^j^, 6 weeks	Physiology, psychological distress
Schuurmans et al [83]	15.25 years, 40.3% F, PTSD	37 40	Muse Passive (TAUk)	unclear, 6 weeks	Physiology
Tarrant et al [84]	41.75 years, 91% F, NT	50 50	BrainLink/virtual reality Active (MAU app)	Body scan, single session	Psychological distress
Vekety et al [59]	9.92 years, 51% F, NT	15 15	Muse Passive	FA/body scans, 4 weeks	Cognitive, brain target

^a^F: female.

^b^MAU app: mindfulness-as-usual with app or audio tracks played at home (self-administered).

^c^FA: focused attention.

^d^NR: not reported.

^e^LF: lowdown focus.

^f^OCD: obsessive-compulsive disorder.

^g^NT: neurotypical; participants had elevated levels of anxiety.

^h^TBI: traumatic brain injury.

^i^PTSD: posttraumatic stress disorder.

^j^OM: open monitoring.

^k^TAU: treatment-as-usual.

### Outcomes of Interest

We extracted a comprehensive set of outcomes from the methods section of the included papers and addressed any missing statistics using reporting bias sensitivity analyses (refer to the *Analysis* section). We collected psychological distress outcomes, including anxiety, depression, fatigue, as well as positive affect, where decreases reflect decreased distress, for example, decreased anxiety or increased positive affect. We collected cognitive outcomes, exclusively behavioral tasks like vigilance and complex reaction time where improvements reflect better cognitive functioning, for example, increased accuracy, or reduced reaction times. We also collected physiological health outcomes including heart rate variability, where increases reflected better health after confirming that these matched hypotheses of the studies. Process variables also were extracted. Specifically, state and trait mindfulness were collected, consisting of established questionnaires like the Five-Facet Mindfulness Questionnaire [85], as well as any study-specific questionnaires that assessed mindfulness as present-moment attention or acceptance. Finally, brain target measures as reported by the consumer-grade devices to assess whether participants were able to successfully modulate brain targets were extracted.

### Systematic Search

A search of PubMed, Web of Science, PsycInfo, and Scopus, was completed on November 11, 2023. Databases were identified based on previous mindfulness systematic reviews and meta-analyses [86,87]. Search terms were “(mindfulness OR meditation) AND (neurofeedback OR neural feedback OR neuro feedback).” We additionally searched reference sections of included papers.

### Study Selection

All studies were first screened for duplicate publications. Next, all abstracts were screened, including studies based on 2 main criteria: an empirical study (examples of excluded articles were review papers and protocol papers) and content relevance (based on above stated eligibility criteria). Then remaining studies were screened by reviewing the methods section and full paper to further evaluate the presence of inclusion criteria. Determination of inclusion was established in cases of disagreement by consulting with the first author.

### Data Collection

A coding manual was developed by the first author to guide the extraction of study descriptive and effect size data. Extraction of these data was conducted by the first author and confirmed independently by authors (ZB, KDG, and NK). Coding disagreements were discussed by the team.

### Data Items

We extracted the following descriptive variables: duration of neurofeedback in minutes and training weeks, participants in mbNF and control conditions (after dropout), control condition type, mbNF device used, delivery modality, type of meditation, age, percentage female (using reported sex), whether or not it was a clinical population, and outcome types. We reported these variables separately for within-participant and between-participant designs (refer to Tables 1 and 2). In the case of multiple intervention groups or control groups, we combined the groups (and pooled their means and SDs). A minority of studies reported overall N but did not report final group numbers, in which case we imputed equivalent group sizes, and coded it as additional risk of bias.

**Table 2:** Overview of all within-participant inductions included in the meta-analysis.

Study identifier	Participant information	Participant number	Control condition	Meditation	Outcome types
Hunkin et al [88]	22.66 years, 58.82% F^a^, NT^b^	35	FA^c^	Muse, FA, 10 min	Brain target, mindfulness
McMahon et al [89]^d^	20.8 years, 20% F, ID^e^	5	FA	Muse, FA, 50 min^f^	Brain target, mindfulness, psychological distress
McMahon [90]^d^	20 years, 50% F, ID	4	FA	Muse, FA, 25 min^f^	Brain target, mindfulness, psychological distress
Sas and Chopra [91]	41 years, 62.5% F, NT	16	unclear	Emotiv binaural/monaural^g^, unclear, 10 min	Brain target, mindfulness
Svetlov [92]	NR^h^, NR, NT	96	FA	Muse, FA, 7 min	Physiology, brain target

^a^F: female.

^b^NT: neurotypical.

^c^FA: focused attention.

^d^These studies involved sequential alternation conditions which are effective replacements for randomization with small samples.

^e^ID: intellectual disability.

^f^Multiple runs.

^g^Binaural or monaural feedback were aggregated.

^h^NR: not reported.

For between-participant designs, we primarily calculated Becker’s del for effect sizes, which is the Cohen *d* for the mbNF group minus the Cohen *d* for the control group [93]. This provides a simple measure of the degree of differential change between the groups. In cases of incomplete reporting, we converted *t* tests or partial eta-squared to Cohen *d.* All effect sizes were converted using Hedges *g* correction, as many sample sizes were small. Variances of effect sizes were calculated using standard methods [93,94]. For within-participant designs, we conducted simple mean differences between the mbNF and control condition, converting using Hedges *g* correction.

### Risk of Bias

Bias assessment was conducted on the RCTs included in the review, following PRISMA (Preferred Reporting Items for Systematic Reviews and Meta-Analyses) guidelines [95]. RCTs were rated using the risk of bias (ROB)-2 [96]. Two authors (IT and either ZB, KDG, or NY) independently rated the risk across several domains, including (1) randomization, (2) blinding, (3) objective measurement of outcomes, (4) attrition, and (5) reporting bias. This estimated risk in each domain was then compared between raters. Any disagreements were discussed and resolved (95% of ratings were identical). After inter-rater agreement was reached, studies were classified as having low, some concerns, or high risk of bias. Methods for quantifying study quality are detailed in Multimedia Appendix 1.

### Analysis

#### Synthesis

Meta-analysis was conducted using the *metafor, MAd,* and *dmetar* packages [97-99]. All measures that met the inclusion criteria were included (ie, psychological distress, cognition, mindfulness, physiological health, or brain target). When studies reported multiple effects (eg, multiple objective measures of cognition), these were first aggregated within each study using the *MAd* package [86]. Aggregating within study ensured that studies with multiple measures (or multiple measures from the same task) did not carry undue weight in the omnibus effect size estimates. For each study, an overall effect size in Hedges *g* units along with a 95% CI was computed.

Omnibus estimates were calculated for psychological distress, cognitive functioning, physiological health, state and trait mindfulness (aggregated jointly), and brain targets; this was done separately for within-participant and between-participant designs. Omnibus estimates were only conducted if there were at least 4 studies reporting an effect [100]. In addition, if the effect did not have a clear hypothesized relationship to the construct, it was not included (eg, is higher or lower resting heart rate beneficial?). Heterogeneity was reported in terms of *I*^2^ and *tau,* and omnibus estimates were classified as low, moderate, or high based on *I*^2^. Analyses used random effect models with study effect sizes weighted by the inverse of their variance, in *metafor*, using restricted maximum likelihood. Random effects models are predominantly used in meta-analyses in psychology [63,87,101] especially when there are reasons to believe that the studies have meaningful differences (eg, samples and devices). We removed any individual studies that showed confidence intervals that did not overlap with the omnibus 95% CI [98]; this only minorly affected results for psychological distress (refer to results). We additionally tested the following moderators: percentage female, age, clinical population (0 or 1), total sample size, neurofeedback duration (for RCTs, days; for within-participant, minutes), and ROB quality of study (refer to Multimedia Appendix 1). We did not conduct moderations by control type, neurofeedback device type or preregistration status, as there were not sufficient studies in each class.

#### Reporting Bias

During effect size extracting, we observed possible reporting bias. Many studies reported nonsignificant effects for a measure and then did not report stats (“class A”). In addition, other studies reported measures in their methods and then did not report stats nor significance (“class B”). We conducted sensitivity analyses to determine if the omnibus effects were sensitive to these classes of missing reporting. Moderate correction consisted of imputing zeros for all effects in class A, and strict correction consisted of imputing zeros for all effects in class A and B. We reported analyses using moderate correction, and noted if the corrections resulted in any differences. As we are analyzing multiple domains of effects, it is appropriate to integrate all relevant reported findings, even if they were not primary a priori hypotheses of the studies. This means that our interpretations based on these sensitivity analyses may be conservative.

#### Publication Bias

The fields of psychology and neuroscience are affected by publication bias (the likelihood that positive results have a higher probability of getting published [102] and so-called “data contingent” analyses [103]). For example, 1 study estimated that psychology’s published findings contain greater than 90% significant results [104]. Such a high percentage of positive findings is statistically highly unlikely especially given widespread low power. It is possible that publication bias affects the field of cgNF. We conducted 2 different approaches, trim-and-fill, which corrects for publication bias in small samples, and 3-parameter selection models which explicitly model the proportion of studies below a *p*-threshold. We considered applying p-curve approaches, but they require at least 3 significant findings which was not the case for multiple models.

## Results

### Study Characteristics

**Figure 2:**
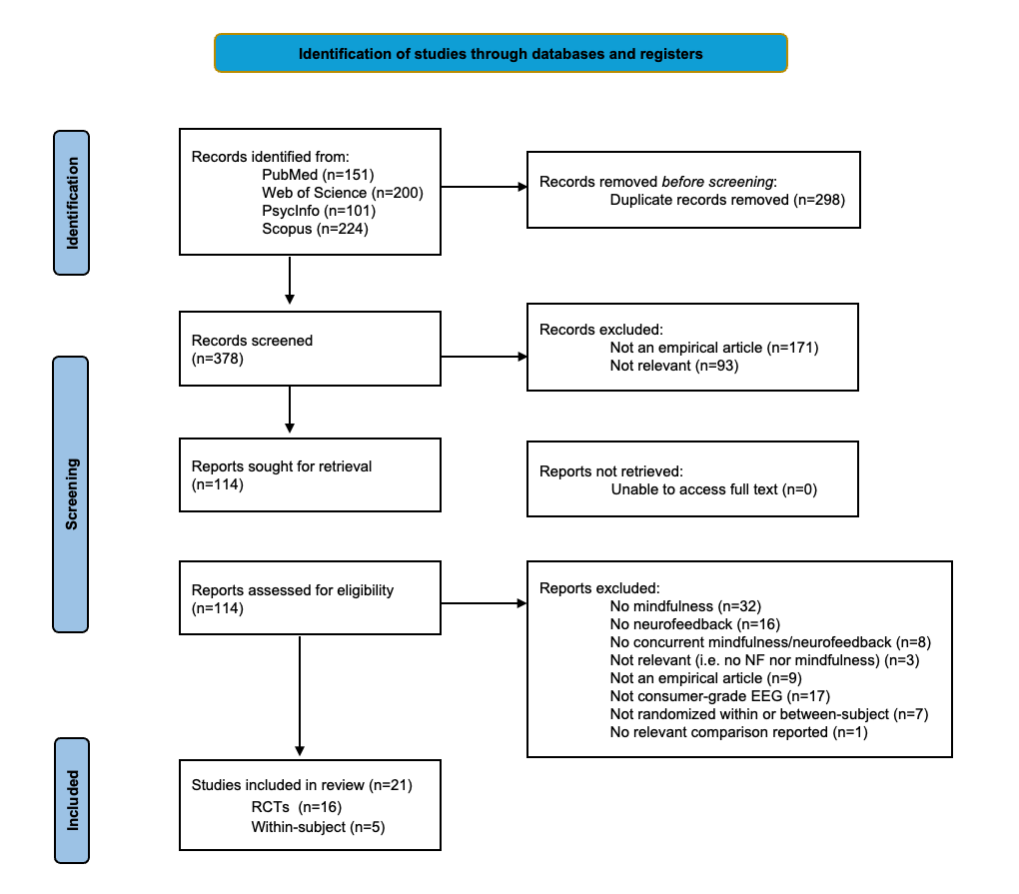
PRISMA (Preferred Reporting for Systematic Reviews and Meta-Analyses) flow diagram, depicting the screening of articles for concurrent mindfulness meditation and consumer-grade neurofeedback procedures [76]. EEG: electroencephalography; NF: neurofeedback; RCT: randomized controlled trial.

A PRISMA flow diagram is shown in Figure 2 (PRISMA checklist provided in Multimedia Appendix 2). A total of 16 RCTs were identified, encompassing 763 unique participants, as well as 5 within-participant randomized studies, encompassing 157 participants. Average sample size was 47.68 participants and 31.2 participants, respectively. The average age was 25.69 (SD 10.01) years and 26.12 (SD 9.99) years, respectively. Three RCTs involved children [66] or adolescents [98,99]. The average proportion female was 66.19% and 47.83%, respectively. Sociodemographic variables were not consistently reported. Four RCTs involved clinical samples (eg, obsessive compulsive disorder [80]), and 2 inductions involved individuals with intellectual disability [89,90]. Most studies used the Muse neurofeedback system (11/16 interventions and 4/5 inductions). The predominant meditation type was focused attention on the breath. Control conditions for studies were primarily active, consisting of mindfulness apps for RCTs (11/16), and short mindfulness meditations for within-participant designs. Passive controls (3 studies) consisted of waitlist or treatment-as-usual. Of the 16 RCTs, 7 were conducted by the same research group in Italy [73,78]. Two of the within-participant randomized studies had extremely small sample sizes (*n*<10). Four studies were preregistered. Full descriptions may be found in Tables 1 and 2.

### Risk of Bias (RCTs)

**Figure 3:**
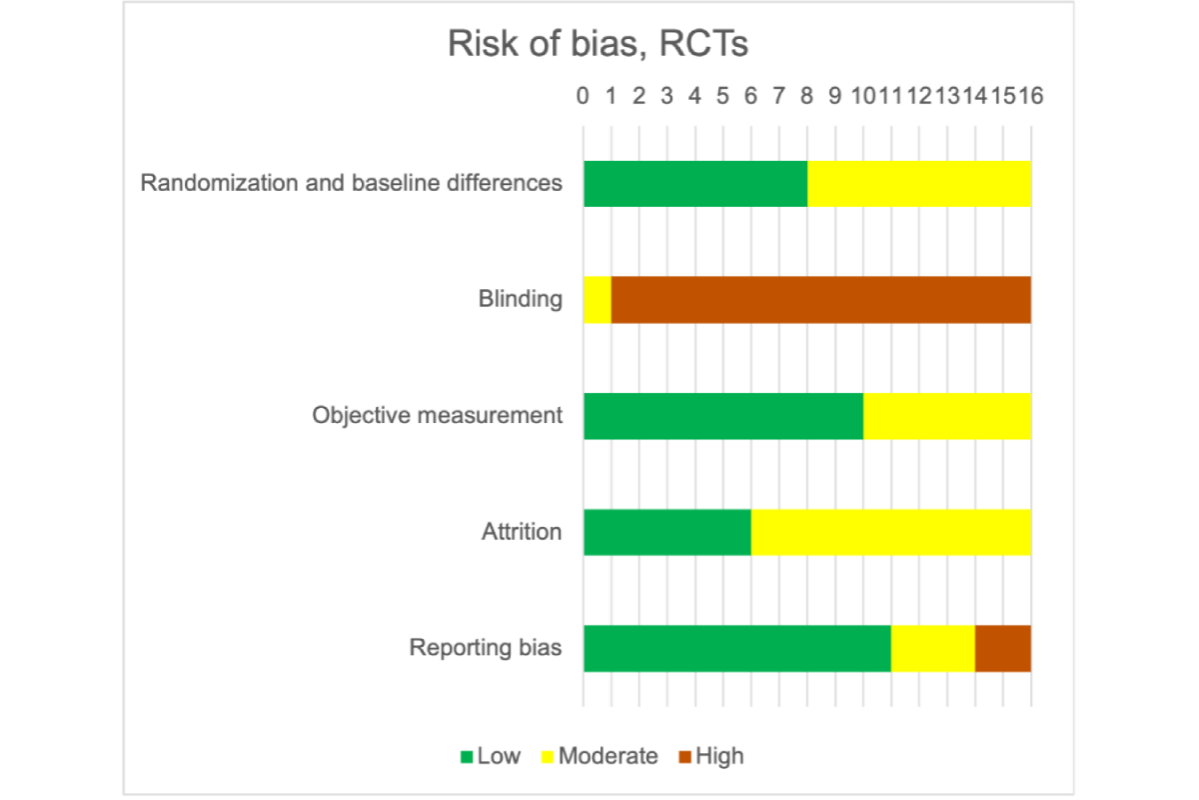
Severity of risk of bias variables for randomized controlled trials included in the meta-analysis. RCT: randomized controlled trial.

The greatest concern in the RCTs was blinding (Figure 3 and Figure S1 in Multimedia Appendix 1). One study had a biofeedback control condition [82], and no studies had sham neurofeedback. There were also concerns with inadequate reporting of randomization and attrition. We address reporting bias in sensitivity analyses.

### Synthesis of RCTs

**Figure 4:**
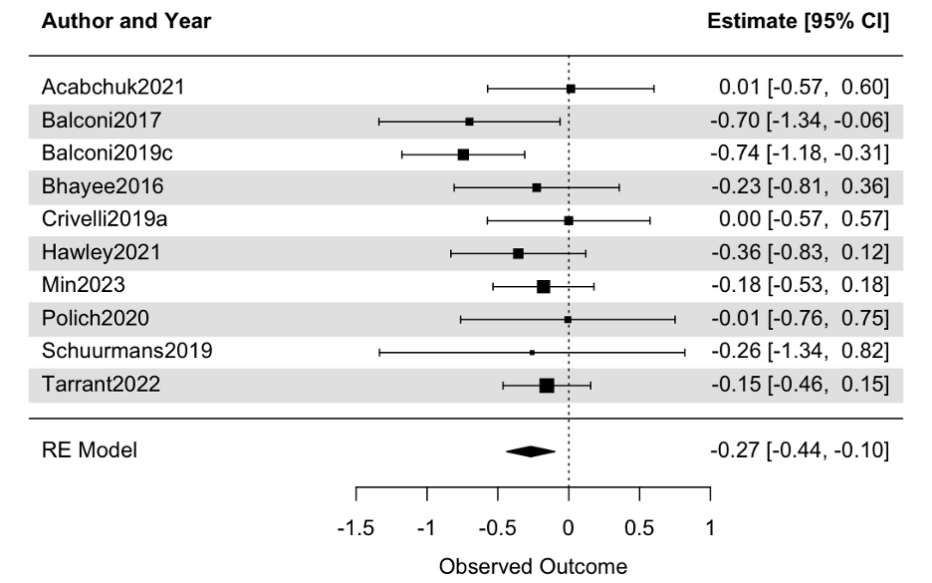
Effects of mindfulness-based neurofeedback on psychological distress within randomized controlled trials. Moderate reporting bias correction was conducted (nonsignificant effects imputed), and one outlier was removed.

**Figure 5:**
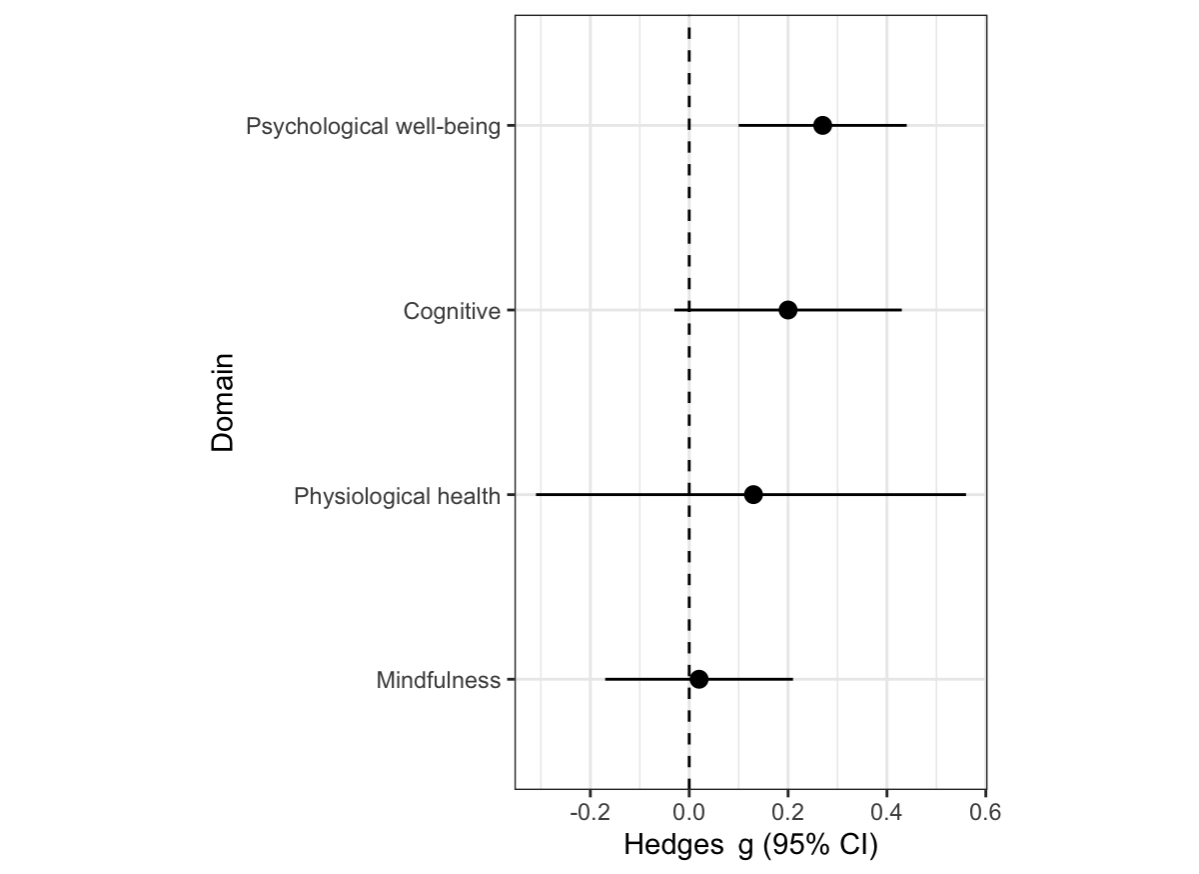
Randomized controlled trial omnibus effect sizes with moderate reporting bias correction. Psychological well-being refers to psychological distress, with effect size reversed for consistency with other outcomes.

Effects for psychological distress were significant across a range of reporting bias corrections (*g*=–0.29 to –0.16, *P*<.05; Tables 3 and 4, Table S2 in Multimedia Appendix 1, and Figure 4). Heterogeneity was low after removing one outlier (*I^2^*<25%). Effects for cognitive function were sensitive to reporting bias, without correction the omnibus effect was significant (*g*=0.31, *P*=.04), but when correcting the effects were not significant (moderate *g*=0.20, strict *g*=0.07, *P>*.10; Tables 3 and 4 and Table S2 in Multimedia Appendix 1). Heterogeneity was moderate for cognitive outcomes (50%>*I^2^*>25%). Effects for physiological health, and mindfulness, were not significant (*P*>.10), and heterogeneity for physiology was high (*I^2^* >75%), even though the measures narrowly focused on heart rate and heart rate variability. Figure 5 presents the omnibus effect sizes for comparison. Results were similar when examining only studies with active controls (Table S3 in Multimedia Appendix 1). In summary, there was limited evidence for decreased psychological distress, but no identified improvements in cognitive function, physiological health, or mindfulness.

**Table 3:** Omnibus effect sizes for randomized controlled trials by outcome and process domains, without reporting bias adjustment.

Domain		Total participants, N	Studies after outlier removal, (outliers, if any, in parentheses)	Effect size (Hedges *g*) (95% CI)	*I*^*2*^ (tau)^a^
Psychological distress		466	9 (1)	–0.29 (–0.47 to –0.11)	16.19 (0.11)
Cognitive		230	7	0.31 (0.01 to 0.61)	42.32 (0.26)
Physiological health		113	4	0.25 (–0.44 to 0.94)	88.9 (0.66)
**Process variable**					
	Mindfulness	310	6	0.08 (–0.15 to 0.31)	0 (0)
**Exploratory**					
	Brain target	165	3	–0.01 (–0.39 to 0.36)	23.18 (0.16)

^a^*I^2^* (tau): heterogeneity measures.

**Table 4:** Omnibus effect sizes for randomized controlled trials by outcome and process domains, with moderate adjustment for reporting bias.

Domain			Total participants, N		Studies after outlier removal (outliers, if any, in parentheses)		Effect size (Hedges *g*) (95% CI)		*I*^*2*^ (tau)^a^
Psychological distress			501		10 (1)		–0.27 (–0.44 to –0.10)		13.21 (0.1)
Cognitive			230		7		0.20 (–0.03 to 0.43)		19.31 (0.14)
Physiological health			321		6		0.13 (–0.31 to 0.56)		77.6 (0.46)
**Process variable**									
	Mindfulness	345		7		0.02 (–0.17 to 0.21)		0 (0)	
**Exploratory**									
	Brain target	—^e^		—		—		—	

^a^*I^2^* (tau): heterogeneity measures.

^b^Nonexistent reporting bias.

There were not sufficient studies to detect whether training increased the brain target scores. However, we conducted an exploratory analysis with the 3 RCTs that reported brain target scores and found no significant effect (*g*=–0.01; *P*=.95; 95% CI –0.39 to 0.36; *I^2^*=23.18; *tau*=0.16). We conducted an exploratory analysis on single-arm effects (no controls), which showed increased brain target scores (Multimedia Appendix 1).

### Synthesis of Within-Participant Designs

We had sufficient studies to analyze whether mindfulness was higher during neurofeedback than during control conditions (*k*=4, *g=*0.14) and whether the brain target was modulated more during neurofeedback than during control (*k*=5, *g*=0.12; Table 5 and Figure S5 in Multimedia Appendix 1). Neither effect was significant (*P*>.15). There was no indication of reporting bias.

**Table 5:** Omnibus effect sizes for within-participant inductions by process domains.

Domain	Total participants, N	Studies after outlier removal, N	Effect size (Hedges *g*) (95% CI)	*I*^*2*^ (tau)^a^
Brain target	157	5	0.12 (–0.1 to 0.34)	25.36 (0.13)
Mindfulness	60	4	0.14 (–0.07 to 0.36)	0 (0)

^a^*I^2^* (tau): heterogeneity measures.

### Moderation

We largely did not find significant moderation effects for clinical population, age, percentage female, quality, or neurofeedback duration (Tables S4 and S5 in Multimedia Appendix 1). Physiological effects correlated negatively with overall sample size (B=–0.02, *P*=.02) such that stronger effects were observed in smaller samples.

### Publication Bias

For RCT studies, there was no indication of publication bias either through trim-and-fill or through selection models (Table S3 in Multimedia Appendix 1). Selection model regressions minimally changed omnibus estimates, except for physiological health, in which the effect size reversed from *g=*0.13 to *g*=–0.13, but neither was significant.

For within-participant studies, trim-and-fill detected low sample size studies with high effect sizes (Table S3 in Multimedia Appendix 1) and accordingly filled in studies on the left of the distribution, resulting in decreased *g*s that remained nonsignificant (Brain target *g*=0.01, Mindfulness *g*=0.06). This assessment should be qualified as one of the studies was a thesis, and not a publication. Selection models failed due to lack of significant *P* values.

### Adverse Effects

Only 2 of the 21 studies evaluated monitored for adverse events, an important measure of the reliability and safety of an intervention. Neither reported any adverse effects.

## Discussion

### Principal Findings

There is increasing interest in technology-supported mindfulness, for promoting scalability, facilitating beginner practice, and increasing motivation and adherence. Here we examined cgNF for supporting mindfulness practice (mbNF). cgNF devices provide metrics of brain function to the user, putatively allowing them to optimize their brain and psychological states. We meta-analytically examined the effectiveness of mbNF in improving psychological and cognitive functioning, and physiological health as well as modulating state mindfulness and brain targets. Included studies consisted of 16 RCT training studies and 5 within-participant randomized studies. There is some evidence that mbNF may reduce psychological distress compared to control conditions, although the effect is small. Improvements in cognitive function were not robust to reporting bias correction, and effects on physiological health were likewise insignificant. There was no evidence for improvements in trait or state mindfulness, which are process measures of the effectiveness of a mindfulness intervention. Likewise, there is not conclusive evidence that participants can learn to modulate brain targets (eg, Muse “Calm” scores). We largely did not find significant moderation effects for clinical population, age, percentage female, study quality, or neurofeedback duration.

Our main finding was the small positive effect of mbNF (*g*=–0.29) on psychological distress, as measured by questionnaires measuring mood, anxiety, posttraumatic stress disorder symptoms, etc. This effect was observed above and beyond control interventions, including mobile meditation apps, although when conducting strict reporting bias correction, the effect shrank to *g*=–0.16. Benefits on stress and distress have been observed in previous reviews of mindfulness and technologically supported mindfulness [9,60,86]. There is little theoretical work on why neurofeedback may benefit mindfulness practice, but mechanisms in the neurofeedback literature generally fall into 2 categories. One, given the correct brain target, neurofeedback may actually lead to facilitated learning and self-regulation [34,35]. For example, in the context of laboratory-based neurofeedback, it is suggested that learning to regulate the default-mode network may lead to enduring changes in self-awareness, alleviating deleterious cycles of self-criticism [105]. Outside of the laboratory, it is less clear what exactly consumer-grade devices target in terms of brain mechanisms, and thus why they should alter psychological states [52]. We did not find evidence that cgNF improves mindfulness nor lead to changes in brain targets more than control conditions. This lack of mechanistic evidence makes it difficult to conclude that mbNF is alleviating psychological distress through brain modulation.

A second, more plausible mechanism is a specific placebo effect called “neurosuggestion” [106]*.* Western societies place a strong emphasis on biological determinants of the mind, and there is widespread trust and enthusiasm for technology and neuroscience. Meditating with cgNF may lead to enhanced motivation, feelings of self-efficacy, and expectations of benefits [107,108]. It is possible that this led to larger decreases in psychological distress compared with control conditions (which were typically potentially less motivating app trainings). The best way to rule out these explanations is through sham-controlled neurofeedback, where participants in the control condition also wear consumer-grade devices but receive “sham” neurofeedback [109]. In this design, the control participants would also experience “neurosuggestion,” and any differential effects must be related to the targeted brain changes. Even in laboratory-based studies, this evidentiary standard is rare (an exception is a sham-controlled RCT that found mbNF participants increased theta oscillations) [13,110]. None of the included consumer-grade studies in the present review used sham controls. One possible approach could be yoked sham, where control participants receive neurofeedback stimuli from another participant (and thus not associated with their own psychological or brain states). Sham controls are fundamental to establishing the efficacy of mbNF.

### Limitations

Effects may have been obscured because of limited power. In general, sample sizes were small (N<50). There was insufficient reporting to investigate the role of intervention fidelity on effects, including differences in device calibration, participant engagement, and adherence. Likewise, our preregistered moderators did not significantly relate to effect sizes, perhaps due to the lack of overall effects, or due to small samples. Per-protocol or dosage analyses could show that some participants experience greater benefits. Relatedly, none of the studies reviewed here extended longer than 8 weeks. An open-label study of Muse over 90 days found positive outcomes, good adherence and 71% of participants wanted to continue using Muse after the period ended [111]. Long-term effects of neurofeedback are unclear, and adverse events are insufficiently monitored in the field. Finally, some of our methods, while standard for meta-analysis, may have obscured effects. To correct for reporting bias, we first contacted authors, and then imputed zeros for mean effects in case of no response. There may have been nonsignificant but trending effects for these measures. We did not find evidence of publication bias, which is present in other biofeedback fields [112].

Limited efficacy of mbNF may arise from technological limitations. First, cgNF relies on dry electrodes on the scalp, which limits signal quality [113]. Signal may be contaminated by muscle movement in many cases and limited by narrow temporal windows of measurement [52]. Second, even given high fidelity measures of underlying brain signals (eg, alpha or beta power), these signals are not straightforward to map to psychological processes like attention [114,115]. Third, there may be serious variability between individuals in these mappings. For example, mind-wandering episodes are predictable within-individuals using whole-brain fMRI measures but the measures show extensive spatial variability between individuals [116].

### Future Directions

To improve the efficacy of mbNF, one approach is to conduct more laboratory-based studies on mechanisms, including multimodal fusion studies where different imaging modalities are combined to assess common signatures of psychological processes. Another approach may be more extensive data collection and algorithm development by device companies. In the face of great individual variability, it may be useful to more extensively calibrate devices (“personalize”) to users before meditation sessions. Currently, users are given short calibration sessions of variable content before meditation, and one assumes that brain signals during calibration are baselines for the meditation session (eg, the goal is to reduce alpha compared to baseline [56]). There is no participant input in the calibration, and calibration procedures are highly variable across devices. If participants could indicate when they feel calmer or more focused, the devices could learn from their responses. In summary, more neuroscientific research on devices may lead to more effective mbNF, and this does not necessarily entail expensive fMRI studies.

A broader challenge is that mbNF assumes that some degree of monitoring during meditation may facilitate mindfulness. Feedback may actually be distracting in some cases [13]. Breath meditation typically emphasizes paying fine attention to the details of the breath and the sensations in various body parts. When attention wanders, one may lose track of the motion of the breath. Or one’s attention may become less precise and the details of the breath become harder to track. Meditation involves learning to notice these changes and develop insight into your mind. If the practitioner is paying attention to an auditory feedback signal instead, they may lose this awareness, and more importantly, lose out on the learning process of watching awareness fluctuate. It may be the case that open monitoring or “mental noting” [105], which allows for attention to shift between the stimulus, sensations, thoughts, etc, is more amenable (we were unable to examine moderation by meditation practice type in the current review). Furthermore, “intermittent” neurofeedback may facilitate awareness and insight without distractions [117]. This is speculative, and more research on qualitative experiences of neurofeedback (especially with advanced practitioners) is necessary. In addition, clear and thorough monitoring of adverse events is recommended.

### Conclusions

The present meta-analysis of mindfulness-based neurofeedback finds evidence for modest decreases in psychological distress compared to controls. This effect may be placebo-related. There is little conclusive evidence for mechanistic engagement, nor improvements in cognition, mindfulness, nor physiological health. Placebo effects of neurofeedback should be taken seriously, as placebo is increasingly considered a meaningful treatment option for some individuals. In terms of treatments, cgNF is relatively inexpensive and accessible, devices are available online for US $100-$200 whereas MBSR courses or therapy or fMRI neurofeedback cost thousands of dollars. However, there are concerns about the commodification of mindfulness which originated as a spiritual practice [118].

Ultimately neurofeedback, biofeedback, and neuromodulation as technological supports for meditation may be constrained by the limits of noninvasive measurement, and the difficulty of linking such limited measures to mental states in a generalizable way. Of course, other forms of technology-supported mindfulness may be more powerful. For example, virtual reality and video games provide immersion, enhance mindful states and may reach people who are not interested in formal practice [9,10,119,120]. Mindfulness apps provide accessibility and customization for specific groups of individuals [121]. However, we should not overlook the significant role that teachers and therapists play in the success of standard mindfulness interventions [66,122-125], and more broadly, the key role of the therapeutic alliance on mental health outcomes even within technology-based treatments [126-129]. Given the escalating mental health crisis, it is imperative for practitioners to thoughtfully integrate evidence-based behavioral interventions with emerging technologies, while maintaining a rigorous focus on understanding which approaches work best for specific populations and the underlying mechanisms that drive their effectiveness.

## Appendix

Multimedia Appendix 1 Additional files.

Multimedia Appendix 2 PRISMA (Preferred Reporting for Systematic Reviews and Meta-Analyses) checklist.

## Abbreviations

cgNF: consumer-grade neurofeedback
EEG: electroencephalography
fMRI: functional magnetic resonance imaging
MBI: mindfulness-based interventions
mbNF: mindfulness-based neurofeedback
MBSR: mindfulness-based stress reduction
PRISMA: Preferred Reporting for Systematic Reviews and Meta-Analyses
ROB: risk of bias
RCT: randomized controlled trial

## Data Availability

Full code and formulas can be found in the provided code [130].
