# Supplementary material for: Consumer-Grade Neurofeedback With Mindfulness Meditation: Meta-Analysis
Source: J Med Internet Res. 2025 Apr 17;27:e68204. doi: 10.2196/68204 (PMC12046271; doi:10.2196/68204)
Supplement: Multimedia Appendix 1 [file jmir_v27i1e68204_app1.docx]

**Supplement: Limited effectiveness of consumer grade neurofeedback with mindfulness meditation : a meta-analysis of controlled designs**

Isaac N. Treves, Zia Bajwa, Keara D. Greene, Nayoung Kim, Emma Wool, Simon B. Goldberg, Susan Whitfield-Gabrieli, Randy P. Auerbach

**Deviations from Preregistration:**

We made the following deviations from the preregistration (https://osf.io/8fz73) :

- For publication bias assessments, we evaluated selection models as well as trim-and-fill in order to test the possibility that effects in specific p-value ranges were being selected for publication. This is increasingly common in modern meta-analysis.
- We did not examine sensitivity to single sessions of training for the RCTs, as there was only one single-session RCT.
- We conducted sensitivity analyses for reporting bias as we observed significant omissions of reporting in many studies.
- For group comparisons, we did not replicate interventions in the case of two controls, instead we pooled the control conditions (if they were both active). This applied in the case of one RCT, Min et al., 2023.

**Quality coding:**

We used the ROB-2 method (Sterne et al., 2019). To generate a continuous estimate of quality, we coded low-risk as 3, moderate risk as 2, and high-risk as 1, and then summed the scores across domains. We then entered quality as a moderator in the random effects models.

**Exploratory analysis, single-arm:**

We had sufficient studies to do a meta-analytic estimate for single-arm (no control) mbNF pre-post changes in brain target scores (Acabchuk et al., 2021; Min et al., 2023; Polich et al., 2020; Vekety et al., 2022). There was an effect, but we observed extremely high heterogeneity (*k* =4, *g* = 0.45, 95% CI [0.01, 0.90], I^2^ =73.71, *tau* = 0.38). This could indicate that mindfulness practice generally increases brain target engagement, but participants were unable to volitionally modulate the target.

| **Section and Topic** | **Item #** | **Checklist item** | **Location where item is reported** |
| --- | --- | --- | --- |
| **TITLE** | | |  |
| Title | 1 | Identify the report as a literature review. | p. 1 |
| **ABSTRACT** | | |  |
| Abstract | 2 | Provide a structured summary including, as applicable: background; objectives; data sources; study eligibility criteria, participants, and interventions; study appraisal and synthesis methods; results; limitations; conclusions and implications of key findings.  See the [PRISMA 2020 for Abstracts checklist](http://www.prisma-statement.org/Extensions/Abstracts.aspx) for the complete list. | p. 2 |
| **INTRODUCTION** | | |  |
| Rationale | 3 | Describe the rationale for the review in the context of existing knowledge, i.e., what is already known about your topic. | p. 4, para. 3 |
| Objectives | 4 | Provide an explicit statement of the objective(s) or question(s) the review addresses with reference to participants, interventions, comparisons, outcomes, and study design (PICOS). | p. 4, para. 5 |
| **METHODS** | | |  |
| Eligibility criteria | 5 | Specify the inclusion and exclusion criteria for the review and how studies were grouped for the syntheses with study characteristics (e.g., PICOS, length of follow-up) and report characteristics (e.g., years considered, language, publication status) used as criteria for eligibility, giving rationale. | Table 1 |
| Information sources | 6 | Specify all databases, registers, websites, organisations, reference lists and other sources searched or consulted to identify studies. Specify the date when each source was last searched or consulted. | p. 7, para. 2 |
| Search strategy | 7 | Present the full search strategies for all databases, registers and websites, including any filters and limits used. | p. 7, para. 2 |
| Selection process | 8 | State the process for selecting studies (i.e., screening, eligibility).  Specify the methods used to decide whether a study met the inclusion criteria of the review, including how many reviewers screened each record and each report retrieved, whether they worked independently, and if applicable, details of automation tools used in the process. | p. 8, para. 1 |
| Study risk of bias assessment | 11 | Specify the methods used to assess risk of bias in the included studies, including details of the tool(s) used, how many reviewers assessed each study and whether they worked independently, and if applicable, details of automation tools used in the process. | p. 8, para. 5 |
| **RESULTS** | | |  |
| Study selection | 16a | Describe the results of the search and selection process, from the number of records identified in the search to the number of studies included in the review, ideally using a flow diagram. | Figure 2 |
|  | 16b | Cite studies that might appear to meet the inclusion criteria, but which were excluded, and explain why they were excluded. | Figure 2 |
| Study characteristics | 17 | Cite each included study and present its characteristics (e.g., study size, PICOS, follow-up period). | pp. 11-14 |
| Risk of bias in studies | 18 | Present assessments of risk of bias for each included study. | Figure 3, Figure S1 |
| Results of individual studies | 19 | For all outcomes, present, for each study: (a) summary statistics for each group (where appropriate) and (b) an effect estimate and its precision (e.g. confidence/credible interval), ideally using structured tables or plots. | Figure 4, Figure S2-S6 |
| **DISCUSSION** | | |  |
| Discussion | 23a | Provide a general interpretation of the results in the context of other evidence. | p. 20 |
|  | 23b | Discuss any limitations of the evidence included in the review. | p. 21, para. 2-3 |
|  | 23c | Discuss any limitations of the review processes used. | p. 21, para. 2-3 |
|  | 23d | Discuss implications of the results for practice, policy, and future research. | Conclusions |
| **OTHER INFORMATION** | | |  |
| Registration and protocol | 24a | Provide registration information for the review, including register name and registration number, or state that the review was not registered. | p. 6, para. 1 |
|  | 24b | Indicate where the review protocol can be accessed, or state that a protocol was not prepared. | p. 6, para. 1 |
|  | 24c | Describe and explain any amendments to information provided at registration or in the protocol. | Supplement p. 1 |
| Support | 25 | Describe sources of financial or non-financial support for the review, and the role of the funders or sponsors in the review. | p. 23 |
| Competing interests | 26 | Declare any competing interests of review authors. | p. 23 |
| Availability of data, code, and other materials | 27 | Report which of the following are publicly available and where they can be found: template data collection forms; data extracted from included studies; data used for all analyses; analytic code; any other materials used in the review. | p. 23 |

**Table S1: PRISMA Checklist.**

*From: Page MJ, McKenzie JE, Bossuyt PM, Boutron I, Hoffmann TC, Mulrow CD, et al. The PRISMA 2020 statement: an updated guideline for reporting systematic reviews. BMJ 2021;372:n71. doi: 10.1136/bmj.n71*

| **Domain** | **N^a^** | **K^b^ (Outliers)** | **ES^c^ (g) [95% CI]** | ***I^2^* (tau)^d^** |
| --- | --- | --- | --- | --- |
| Psychological Distress | 539 | 11 (1) | -0.16 [-0.31, -0.021] | 0 (0) |
| Cognitive | 232 | 7 (1) | 0.072 [-0.13, 0.27] | 0 (0) |
| Physiological Health | 321 | 7 | 0.11 [-0.26, 0.47] | 72.9 (0.41) |
| **Process Variables** |  |  |  |  |
| Mindfulness | 385 | 9 | 0.02 [-0.16, 0.20] | 0 (0) |

**Table S2:** Strict reporting correction omnibus effects. Domains of outcomes are shown on left. Adjusted effects on right are after strict reporting bias correction (all non-reported measures are imputed).

^a^ N: total number of participants

^b^ K: number of studies after outlier removal (outliers in parentheses)

^c^ ES: effect size, in Hedge’s *g*

^d^ *I^2^* (tau): heterogeneity measures.

| **Domain** | **N^a^** | **K^b^ (Outliers)** | **ES^c^ (g) [95% CI]** | ***I^2^* (tau)^d^** |
| --- | --- | --- | --- | --- |
| Psychological Distress | 432 | 9 (1) | -0.26 [-0.45, -0.07] | 21.48 (0.02) |
| Cognitive | 205 | 6 | 0.22 [-0.04, 0.49] | 31.06 (0.03) |
| Physiological Health | 244 | 5 | 0.15 [-0.40, 0.70] | 79.64(0.29) |
| **Process Variables** |  |  |  |  |
| Mindfulness | 276 | 6 | 0.01 [-0.22, 0.23] | 0 (0) |

**Table S3:** Active controls omnibus effects. Domains of outcomes are shown on left. Adjusted effects are after moderate reporting bias correction (all non-significant measures are imputed).

^a^ N: total number of participants

^b^ K: number of studies after outlier removal (outliers in parentheses)

^c^ ES: effect size, in Hedge’s *g*

^d^ *I^2^* (tau): heterogeneity measures.

|  | Psychopathology | | Cognitive | | Mindfulness | | Physiology | |
| --- | --- | --- | --- | --- | --- | --- | --- | --- |
| Moderator | Beta | P-value | Beta | P-value | Beta | P-value | Beta | P-value |
| Sex | 0 | 0.39 | -0.01 | 0.44 | 0 | 0.97 | -0.01 | 0.25 |
| Age | 0.02 | 0.24 | 0 | 0.85 | 0 | 0.96 | -0.03 | 0.22 |
| Sample Size | 0 | 0.55 | 0 | 0.75 | 0 | 0.67 | -0.02 | 0.02* |
| Duration | 0 | 0.82 | 0 | 0.3 | 0 | 0.7 | 0 | 0.16 |
| Clinical | 0.22 | 0.4 | -0.21 | 0.62 | 0.07 | 0.71 | -0.68 | 0.06 |
| Quality | 0.078 | 0.25 | -0.1 | 0.13 | 0.02 | 0.79 | -0.28 | 0.16 |

**Table S4:** Moderators for interventions. Shown for moderate reporting bias correction (non-significant results imputed). Beta: unstandardized beta. P-value: uncorrected *p*-value. * , *p* < 0.05

|  | Brain Target | | Mindfulness | |
| --- | --- | --- | --- | --- |
| Moderator | Beta | P-value | Beta | P-value |
| Sex | 0 | 0.79 | -0.01 | 0.32 |
| Age | 0.01 | 0.49 | 0.01 | 0.57 |
| Sample Size | -0.0043 | 0.06 | -0.01 | 0.24 |
| Duration | 0.02 | 0.14 | 0.01 | 0.26 |
| Clinical | 0.4 | 0.28 | 0.3 | 0.4 |

**Table S5:** Moderators for inductions. No reporting bias correction (not applicable). No quality score was available (not RCTs). Beta: unstandardized beta. P-value: uncorrected *p*-value.

| **RCT Domain** | **TRIMFILL add studies** | **ES_adj** | **Selection_adj** | **Test of Publication Bias** |
| --- | --- | --- | --- | --- |
| Psychological Distress | No change | No change | -0.25 [-0.46, -0.04] | *p* = 0.85 |
| Cognitive | No change | No change | 0.16 [-0.09, 0.42] | *p = 0.69* |
| Physiological Health | No change | No change | -0.13 [-0.51, 0.26] | *p* =0.20 |
| Mindfulness | No change | No change | No significant effects | No significant effects |
| **Induction Domain** |  |  |  |  |
| Brain Target | 3 | 0.01 [-0.17, 0.18] | No significant effects | No significant effects |
| Mindfulness | 2 | 0.056 [-0.13, 0.24] | No significant effects | No significant effects |

**Table S6:** Publication bias adjustments. Domains of outcomes are shown on left. Column 1: Trim-fill studies added. Column 2: change in omnibus effect size. Column 3: selection model effect size. Column 4: selection model test for publication bias.


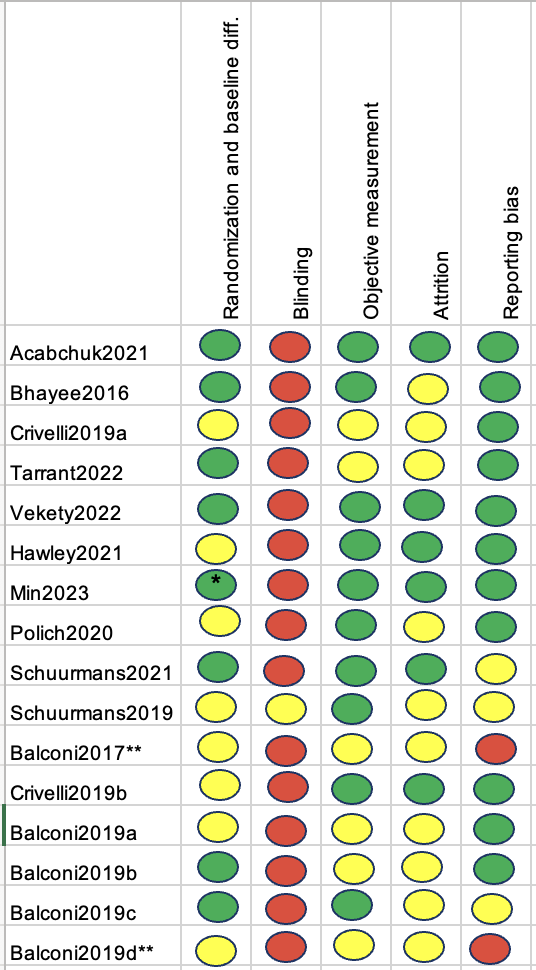


**Figure S1:**

Risk of bias. Red: high risk, Yellow: moderate risk, Green: low risk. * allocation concealment was preserved, ** book chapters.


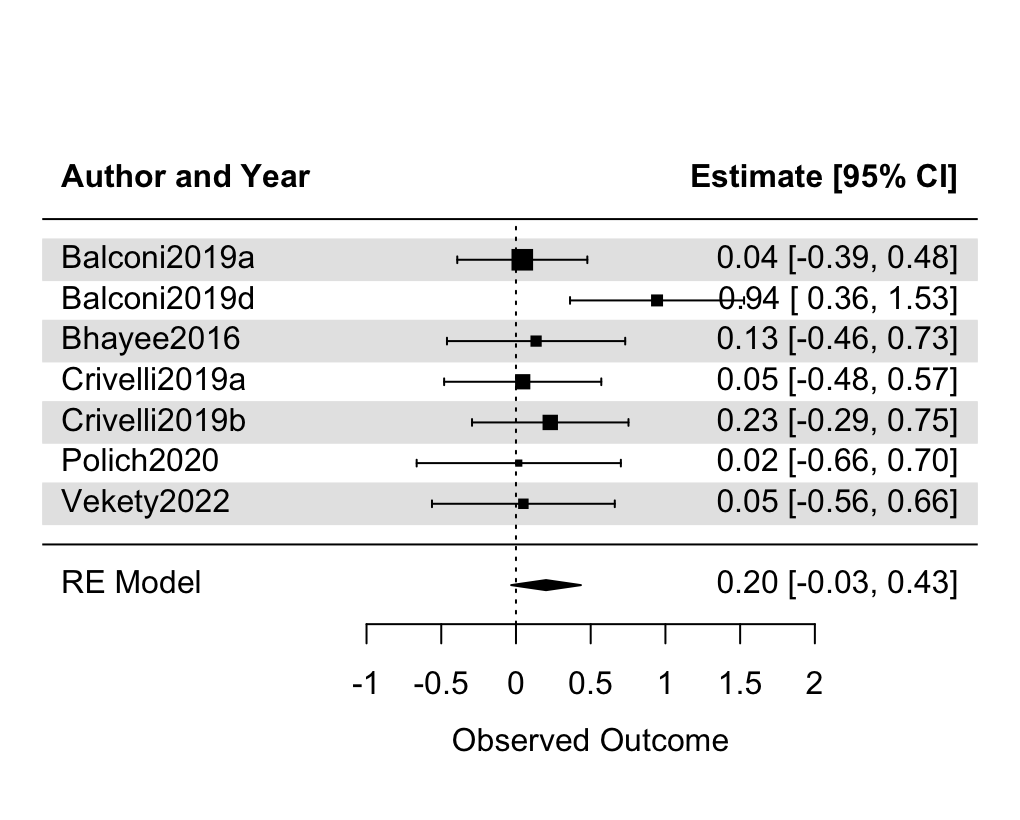


**Figure S2:**

Effects of mbNF on cognitive outcomes, randomized controlled trials. Moderate reporting bias correction was conducted (non-significant effects imputed).


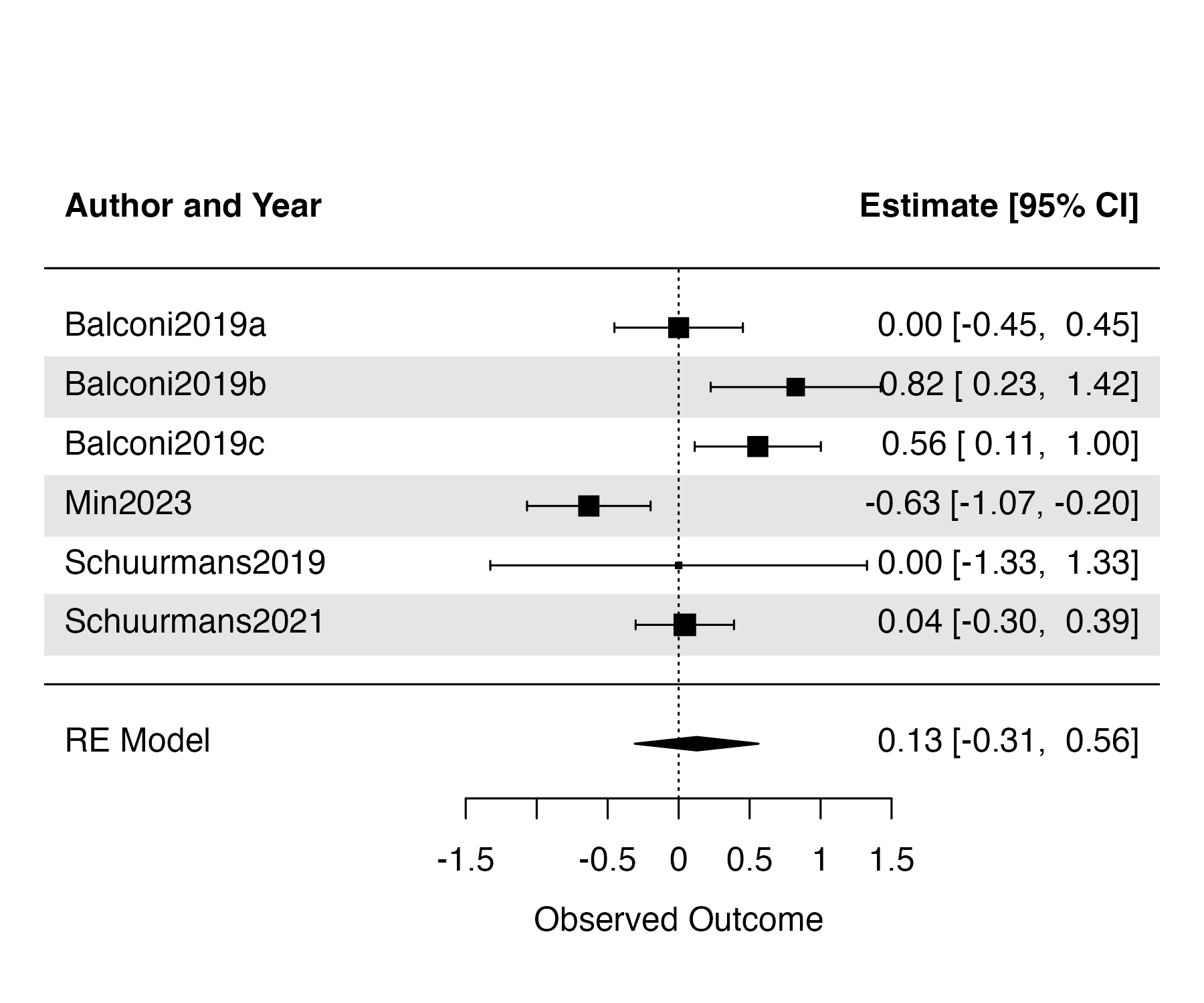


**Figure S3:**

Effects of mbNF on physiology, randomized controlled trials. Moderate reporting bias correction was conducted (non-significant effects imputed).


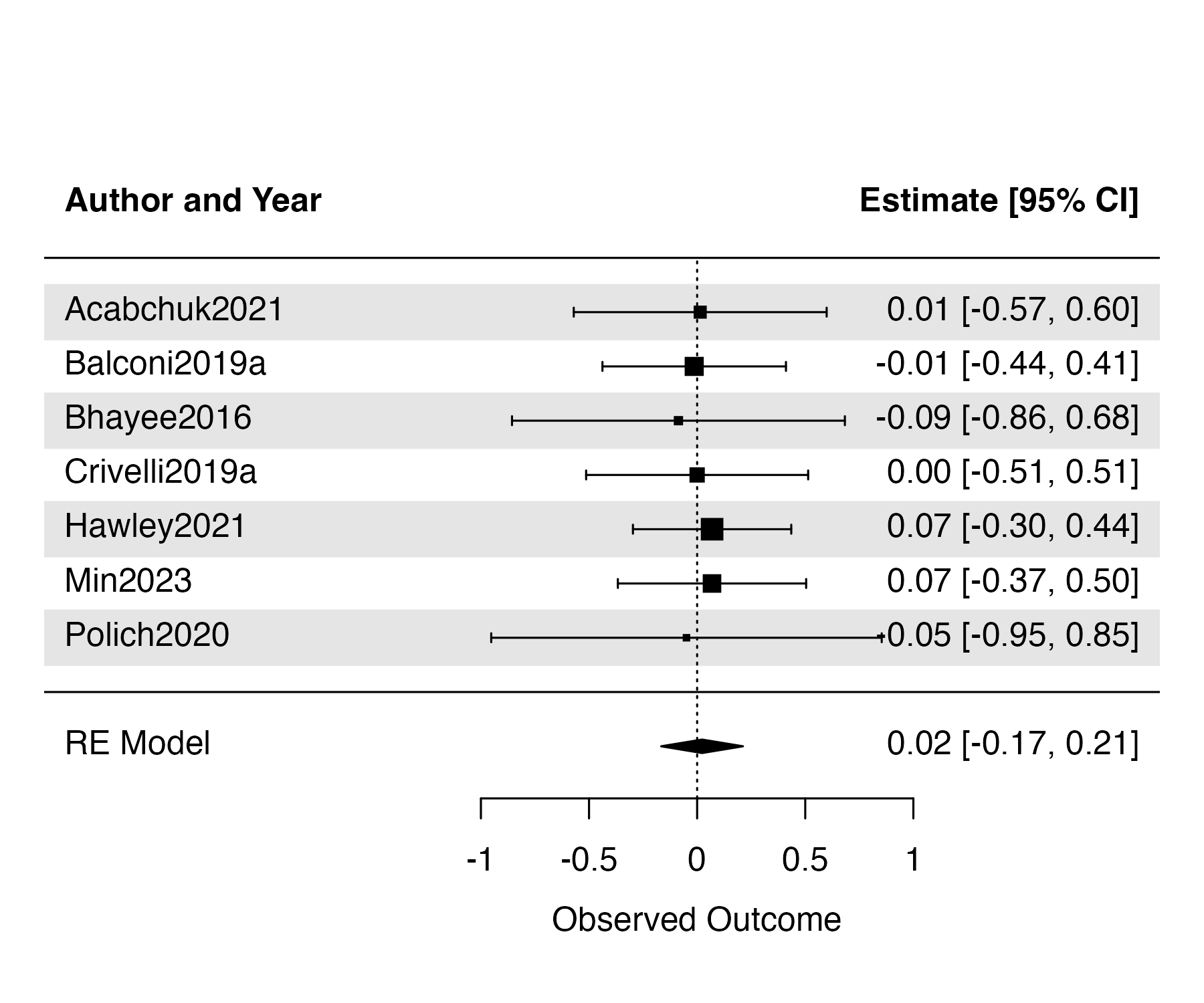


**Figure S4:**

Effects of mbNF on mindfulness, randomized controlled trials. Moderate reporting bias correction was conducted (non-significant effects imputed).


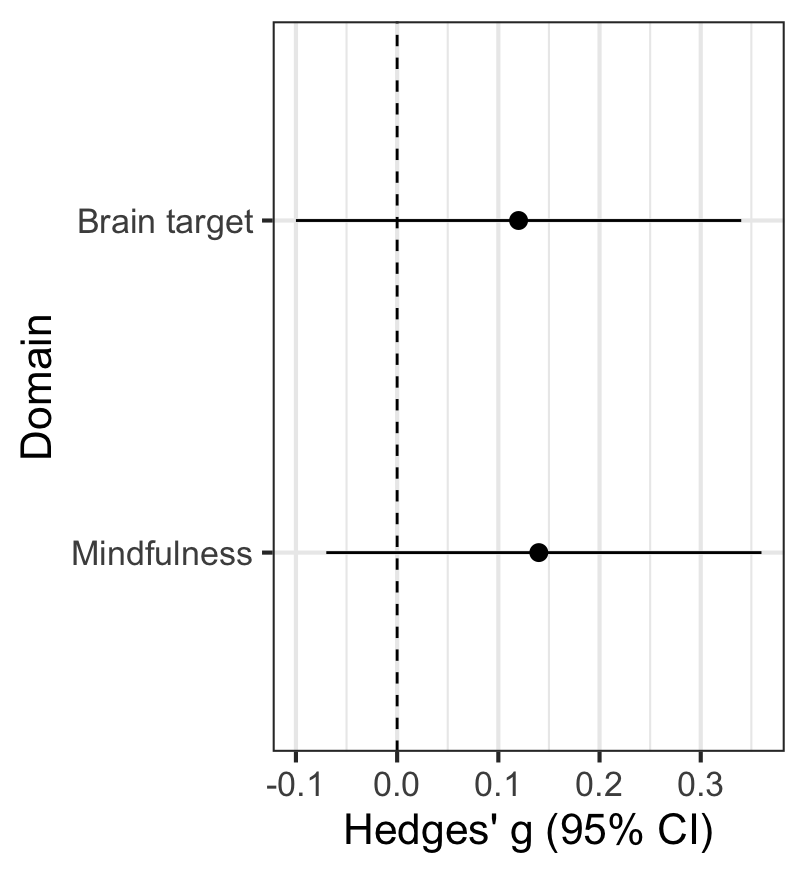


**Figure S5:** Omnibus effect sizes for within-subject inductions.


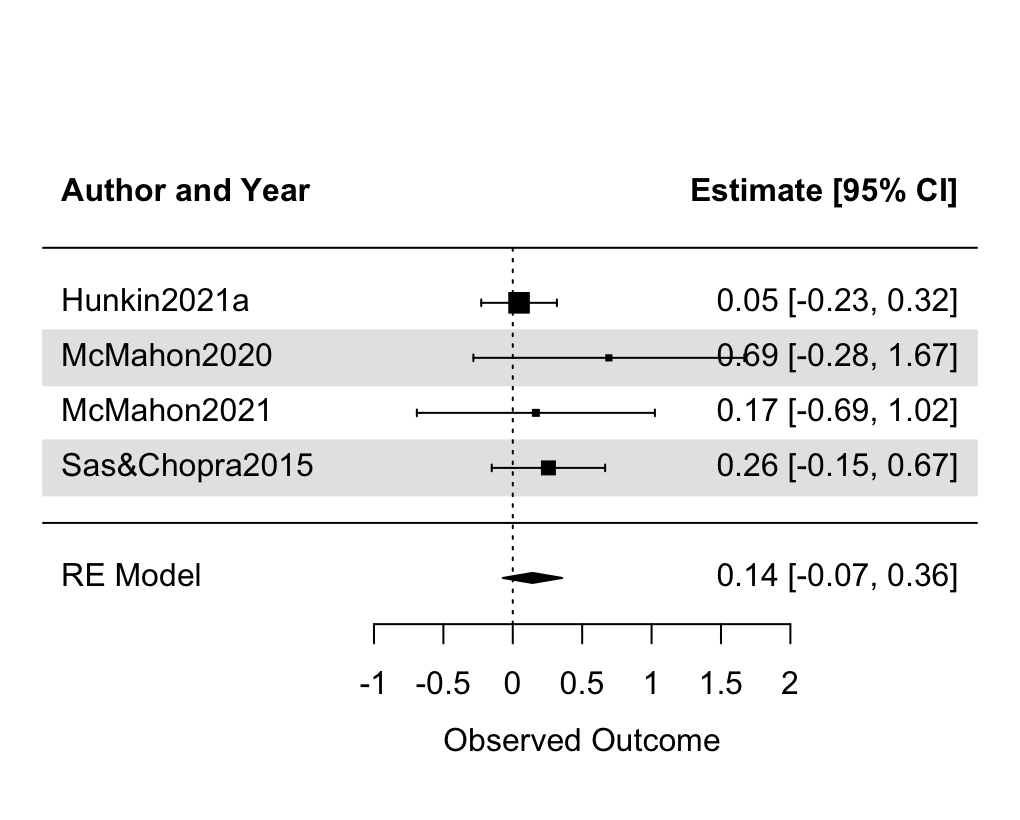


**Figure S5:**

Effects of mbNF on mindfulness, inductions. No reporting bias was identified.


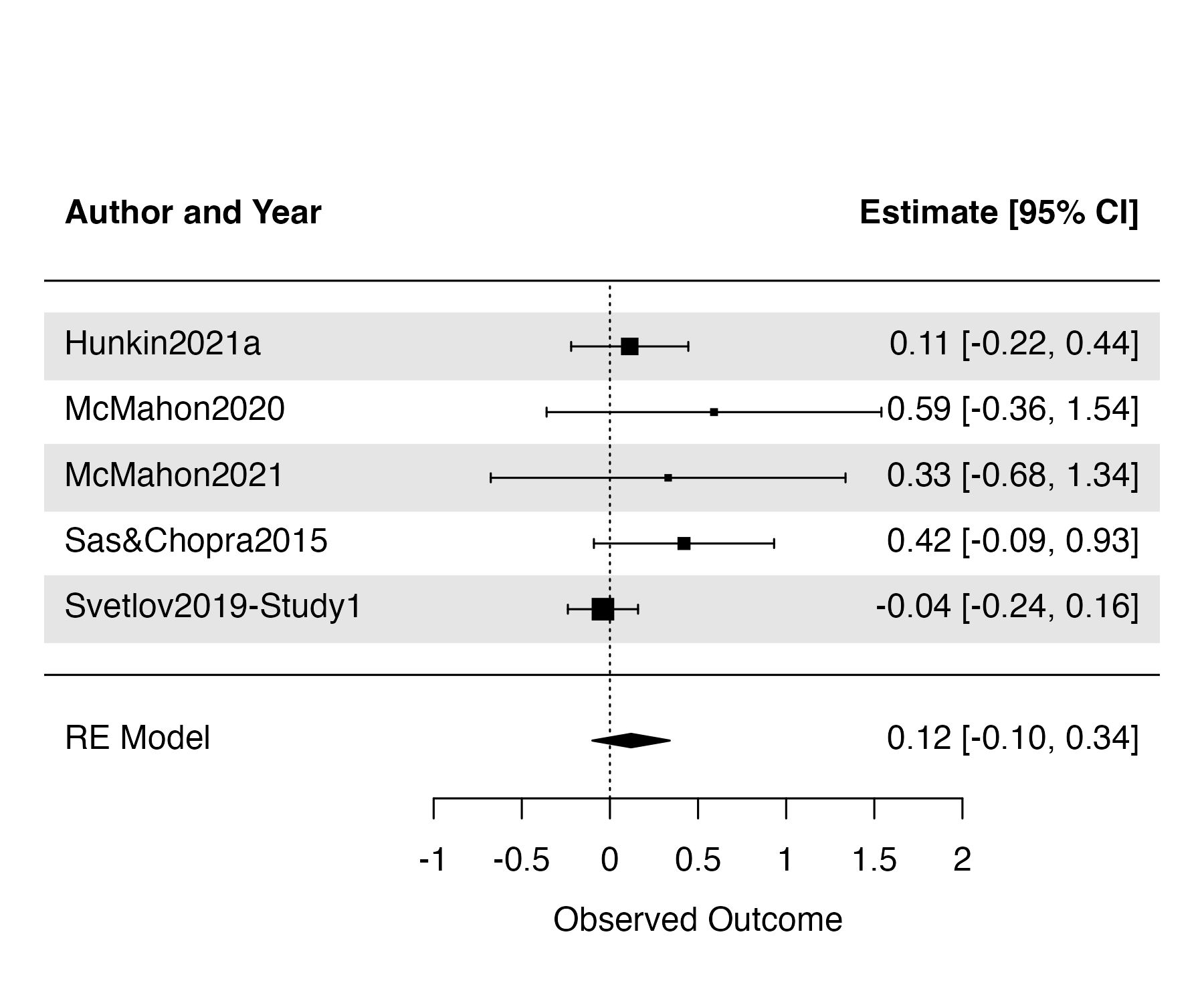


**Figure S6:**

Effects of mbNF on brain targets, inductions. No reporting bias was identified.
